# Cultural Transmission of Traditional Knowledge in two populations of North-western Patagonia

**DOI:** 10.1186/1746-4269-4-25

**Published:** 2008-12-15

**Authors:** Cecilia Eyssartier, Ana H Ladio, Mariana Lozada

**Affiliations:** 1 Inibioma-Universidad Nacional del Comahue, Lab. Ecotono, Quintral 1250–8400 San Carlos de Bariloche, Rio Negro, Argentina

## Abstract

**Background:**

In the present study we have investigated the cultural transmission of two types of traditional plant knowledge in two communities of North-western Patagonia, Argentina. In the Pilcaniyeu community, we studied the transmission of traditional knowledge related to horticultural practices in home-gardens, greenhouses and gardens; while in the community of Cuyin Manzano, we studied wild plant gathering customs.

**Methods:**

Ethnobotanical fieldwork was conducted by means of semi-structured interviews, in which we investigated which plants are used, at what life history phase was learned, modes of transmission and who the principal transmitters were in childhood and adulthood. In both communities, each of this three aspects related to cultural transmission were categorized and the frequencies of each category were obtained. The total number of species recorded in each community was also calculated. Frequencies were analyzed with the Chi-square test of independence.

**Results and discussion:**

In both communities, transmission of traditional plant knowledge begins at an early age, as a family custom, in which women play a predominant role. Wild plant use and horticultural knowledge continue to be learned during adulthood. This was particularly registered associated with horticultural learning, which receives greater influence from extension agents who are introducing new practices and technology. This outside influence, which implies novelty, could imply syncretism but also traditional knowledge loss.

**Conclusion:**

Given the remarkable acculturation processes occurring at present in rural communities of Northwestern Patagonia, it might be of vital importance to document traditional knowledge of ancient practices. Moreover, it could be interesting to share our results with both populations in order to encourage participatory activities within the communities which could enhance traditional knowledge horizontal transmission, particularly among elder adults and youngsters.

## Background

Traditional ecological knowledge (TEK) entails intricate integration between human beings and their natural resources. This construct has been defined as the knowledge acquired by local communities through history, by means of direct experience and contact with nature [1]. It comprises a wide spectrum of rural inhabitants' lives, which include material, spiritual and cultural traits such as wild plant knowledge or traditional agriculture. This cumulative body of knowledge and beliefs evolves by adaptive processes and is handed down through generations by cultural transmission [2]. Therefore, this knowledge is a dynamic process, which generally responds in a flexible way to environmental and socio-cultural changes [3].

Cultural transmission is a process of social dissemination in which behaviour patterns, cosmological beliefs, the culture's technological knowledge, etc. are communicated and acquired [4]. This way of transmission is not simple, and it depends on many factors, such as age, gender and other socio-cultural factors [5]. Cultural transmission occurs between individuals of different generations but within genealogy (vertical transmission), as is the case from parent to child [6]; and it may also occur between individuals of the same generation, irrespective of their relationship (horizontal or contagious transmission). It has been found that vertical transmission is highly conservative. That is why, with this type of transmission, innovations might be very slow to spread in the population unless other modes of cultural transmission are also employed. In the case of "one-towards-many" mode of transmission, communication is highly efficient and cultural change may be quite rapid. On the other hand, in "many-towards-one" way of transmission, an individual is assumed to be influenced by many transmitters, and all transmitters act in concert so that social influence is reciprocally reinforced [4].

Paths of cultural transmission can change during lifetime. It is well known that parents, who share their context with their children, play a crucial role in transmitting knowledge in early life [e.g. [4,7]]. However, as time goes by, peers, who might come from different scenarios, become more significant. In addition, cultural learning also continues throughout adulthood, as individuals interact with new social contexts; an interesting issue which has received little attention. Furthermore, aside from the importance of social-dependent contexts in cultural transmission, individual idiosyncratic factors are also relevant in this acquisition process, most of which derive from personal experiences that promote active filtering of cultural input [7].

Local communities such as the Mapuche have a particular concept of nature which could contribute to the development of sustainable management practices, promoting biological and socio-cultural diversity [2]. There is an increasingly comprehensive appreciation of this knowledge and biodiversity conservation projects have been more successful when local knowledge was considered [8-11].

The Mapuche people were ancient inhabitants of the South Andean region, who lived as hunter-gatherers and semi nomadic horticulturists, in isolated and independent communities [12]. Since the Spanish conquest, these populations have been undergoing severe changes, which dramatically modified their living conditions and caused processes of erosion of their cultural heritage [13-16]. Among other customs, their health system, diet and horticultural knowledge greatly changed, altering their gathering and cultivating experience. In spite of these changes, the Mapuche people still maintain wild plant use and horticultural practices as important aspects of their cultural traditions.

Traditional ecological knowledge related to wild plant use has suffered significant transformations, such as the incorporation of exotic medicinal and edible species [17]. At present, this gathering practice tends to be declining over generations [18,19]. Young people tend to leave their homes, abandoning their ancestral customs as they refocus their interests on the demands of western culture [15]. In relation to their horticultural tradition, other changes have occurred. Native crops such as maize, quinoa, potatoes, pumpkin and pepper have decreased in use, while barley, wheat and oats have been included. Nowadays, their horticultural practices are influenced by extension agent institutions such as INTA (National Institute of Agricultural Technology), PSA (Agricultural Social Program) and CIESA (Center of Investigation and Teaching of Sustainable Agriculture) which have introduced new crops and technologies. In contrast, wild plant gathering does not seem to be influenced so much by these extension institutions.

In the present study, we have compared the cultural transmission of two types of traditional ecological knowledge in two communities of North-western Patagonia. In the Pilcaniyeu community, we studied the transmission of traditional knowledge related to horticultural practices in home-gardens, greenhouses and gardens, and compared with wild plant gathering in the Cuyin Manzano community [20]. Whereas, wild plant gathering is a frequent custom among these rural inhabitants and has been thoroughly studied [15,16,18], horticultural practice has not been previously explored in the region. Both traditions had been historically important for sustenance and are still scenarios for transmission of ancient knowledge, that is why the comparison between both could shed interesting information. We have analysed the actors involved in social networks associated with this learning process, investigating at what life history phase plant knowledge is learned, modes of transmission, and who the principal transmitters were in childhood and adulthood.

## Methods

### Study sites

**Figure 1:**
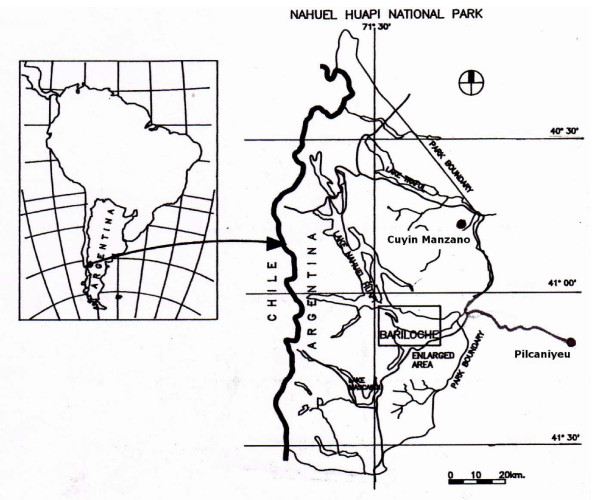
Map of the study sites including Pilcaniyeu and Cuyín Manzano communities.

The Pilcaniyeu community is located in the province of Rio Negro, Argentina (41° 7'S and 70° 44'W) [Fig. 1], and has approximately 1445 inhabitants. Pilcaniyeu's nearest urban population is Bariloche, located 75 km away. The area has a mean annual temperature of 7.3°C and a mean annual precipitation of 264.80 mm. It is characterized by the Patagonian steppe ecosystem, where sandy soils are dominant and the vegetation cover is mostly composed of shrubs and herbs: neneo (*Mulinum spinosum*), charcao (*Senecio filaginoides*); coirón amargo (*Stipa humilis*, *Stipa speciosa*) and *Poa huecu, Bromus macranthus*, *Poa ligularis*, *Festuca argentina *and other herbs [21]. It is also characterized by valleys and swamp areas, with rocky patches in other sectors. The economic sustenance of most inhabitants is cattle-raising, but some work as employees of public institutions and practice horticulture as well. Since 1992, extension agents such as INTA, have been in charge of promoting family, community and school home-gardens in Pilcaniyeu, Ñorquinco and Bariloche. They have been working with rural residents to help agro-systems and encourage socioeconomic development in their communities. In order to promote horticulture, extension agents have introduced new technology, practices and knowledge. As an example of new technology, they have provided the materials to build greenhouses and tools necessary for horticultural practice. These were considered as new technology, given that greenhouses were not traditionally developed by their ancestors. Furthermore, they periodically distribute seeds of exotic plants such as lettuce, carrot, chard, melon, etc. and visit the people to provide assistance according to their agricultural needs.

The Cuyin Manzano is an small rural community made up of 18 families, which is located in the Nahuel Huapi National Park (40° 45 S and 71° 10 W), Neuquén, Argentina, 80 km away from Bariloche city [Fig. 1]. This location is in a transition forest, dominated by a conifer species, *Austrocedrus chilensis*, with the presence of the deciduous species *Nothofagus pumilio*, at higher elevations and *N. antarctica *in the underbrush and scrubs. This area has a mean annual temperature between 5.4° and 9.5°C. Cattle-raising and handicrafts are the principal economic activities of most inhabitants.

Both populations are of mestizo origin, some inhabitants are direct descendants of the Mapuche people, while others have mixed ancestry. At present Pilcaniyeu and Cuyín Manzano have both lost their traditional Mapuche roots, i.e., the inhabitants do not speak the Mapuche language (Mapudumgun), and they have lost their political leaders (caciques) and shamans (machis) [20]

Cuyín Manzano's social organization is weak; the primary school is the most significant communal structure. Pilcaniyeu, on the other hand, shows a more intricate social and political organization. There are several communal structures such as primary and secondary schools, a Town Hall, a local Hospital, and other facilities related to cultural, social, recreational and community development.

### Ethnobotanical data collection

Ethonobotanical information was compared using data from fieldwork done in Pilcaniyeu and from a previous study done in Cuyin Manzano (see [20]). Both ethnographic studies are really comparable given that all methodological procedures were followed in the same way, in spite of the fact that the topics surveyed differed. With the intention of developing the proposed objectives, a formal presentation was initially made to the authorities of each community to request their consent to work in the area. Moreover, in each visited domestic unit, we asked for voluntary participation in our interview. Ethnobotanical fieldwork was conducted by means of semi-structured interviews in the summer of 2001 in Cuyín Manzano and in the summer of 2007 in Pilcaniyeu. In both communities, participants were randomly selected, each representing a different family. A total of 30 people (20 women and 10 men) between 18 and 85 years old, were interviewed in the Pilcaniyeu community (approximately representing 20% of the total number of families), specifically those who dedicate greater time to horticultural practices. A total of 16 individuals (7 women and 9 men) between 27 and 74 years old were interviewed in Cuyín Manzano (representing 80% of the total number of families). The semi-structured interviews, which lasted approximately 60 to 90 minutes, were based on general aspects of the interviewee's daily life in relation to plants. In the community of Pilcaniyeu, we asked about cultivated plants and traditional horticultural practices, while in the community of Cuyin Manzano we investigated wild plant knowledge and gathering practices. The interviewees' personal data, such as gender and age, was gathered as well. We were also interested in aspects related to cultural transmission of traditional knowledge: at what age and how plant knowledge was learned and who the principal transmitters were.

In order to prepare field herbariums, wild plants and cultivated species were collected in both communities with the participation of the inhabitants which were visited in a second opportunity. The recorded registers, field notebooks, photographic material and field herbariums were deposited in the Ecotono Laboratory, Centro Regional Universitario Bariloche. Nomenclature follows Correa [22], Marticorena & Quesada [23] and Ezcurra & Brion [24].

### Data Analysis

In both communities, the following aspects related to cultural transmission were categorized: at what life history phase plant knowledge was learned, modes of transmission and who the principal transmitters were in childhood and adulthood. In turn, the frequencies of each category for each question were obtained. Additionally, in order to quantify the richness related to both practices, we calculated the total number of species recorded in each community. The frequency of these categories was analysed with the Chi-square test of independence (p < 0.05) [25].

## Results

### Modes of transmission in childhood

**Figure 2:**
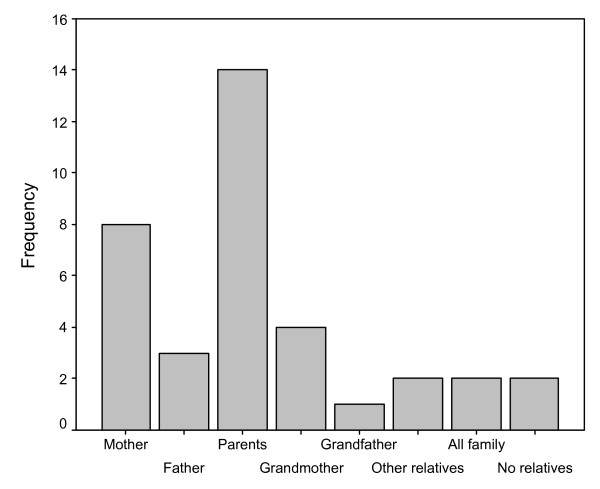
Transmitters of traditional ecological knowledge related to horticultural practices in chilhood in Pilcaniyeu community.

In the Pilcaniyeu community, the dwellers mentioned different transmitters of traditional knowledge related to horticultural practices and that they have learnt about these practices since childhood. When we compared the categories mentioned, significant differences were found among their responses (X^2^_5 _= 16.424, p < 0.05) (Fig. 2). Both parents were mentioned as main transmitters of this type of knowledge (39%). However, mothers seem to be important transmitters since they were mentioned in second place (22.2%); followed by grandmothers (11.1%). On the other hand, in 8.3% of the cases, learning was cited from their fathers. In only one case, a grandfather was mentioned as the first transmitter (2.8%). Additionally, in 5.5% of the cases other relatives (aunt, mother-in-law, etc.) were mentioned. The whole family was also mentioned as the source of learning in 5.5% of the cases. Finally, the same proportion was recorded for extra-family learning (teachers, friends, etc.).

**Figure 3:**
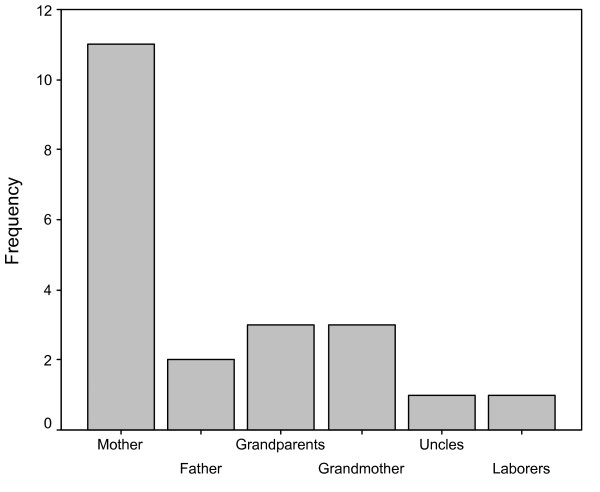
Transmitters of traditional ecological knowledge related to wild plant gathering practice in childhood and adulthood in Cuyin Manzano community.

In Cuyín Manzano, the informants mentioned different people as transmitters and that they have learnt about wild plant gathering practice in childhood as well. When these categories were compared, significant differences were found (X^2^_4 _= 17.81, p = 0.001) (Fig. 3). This traditional practice seems to be mainly transmitted by interviewee's mothers, and grandmothers were specifically mentioned by 50% of the cases when grandparents were cited [20].

### Modes of transmission in adulthood

**Figure 4:**
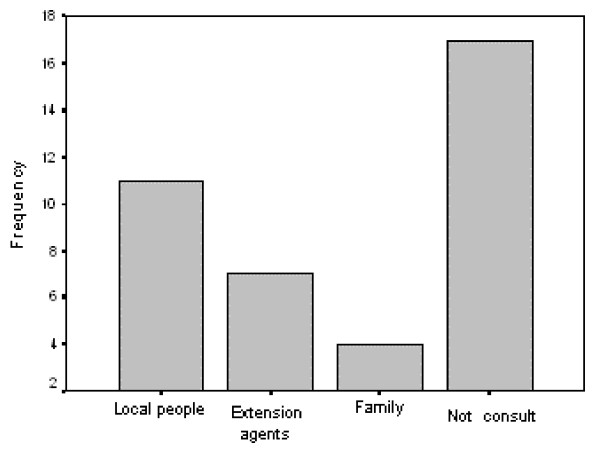
Transmitters of traditional knowledge related to horticultural practices in adulthood, in Pilcaniyeu community.

Significant differences were found in terms of who the main transmitters were in this analysis in the community of Pilcaniyeu (X^2^_3 _= 12,939, p < 0-05) (Fig. 4). In 43.5% of the cases, we observed that adult informants do not consult other people, which means that they learn about horticulture through personal experience. Local people ("locals") represent an important source of learning (28%). This category corresponds to those inhabitants of Pilcaniyeu with vast experience in horticultural practices, an inherited tradition in their families, who have lived in the region for a long time. Extension agents, such as INTA or PSA, were also mentioned as important transmitters, but in a smaller proportion (18%, Fig. 4). They have introduced new practices and technology, such as the use of greenhouses, which differ from the traditional knowledge of local people. Finally, in 10% of the cases, adult informants consult their family.

More than half of the families (68.6%) have incorporated the use of greenhouses. Sixty percent of the informants received help from extension agents (PSA and INTA), while the other 40% built their greenhouses by their own means, with the aid of other family members, neighbours, etc. It seems that this new practice is spread by horizontal transmission among local dwellers.

Traditional knowledge related to wild plant gathering practice in the community of Cuyin Manzano is acquired mostly through vertical transmission which prevails throughout informants' lives. Horizontal and oblique (one-toward-many or many to one) modes of transmissions were not documented in this community [20] (Fig 3).

### Plant species utilized and common uses in both populations

In Pilcaniyeu, 124 ethnospecies were recorded: 75 in home-gardens, 63 in greenhouses and 68 in gardens. Most species (113 spp.) were exotic, which means that they came from other biogeographic regions; while 11 were native to Patagonia. It is important to mention that some of these native species, such as *Chenopodium ambrosioides *and *Anarthorphyllum rigidum*, are not specifically cultivated but tolerated as part of the cultivated areas [see Additional file 1]. Most species were cultivated for edible purposes (home consumption) (41%); whereas 28% were mentioned for ornamental purposes and 20% were cited for medicinal use.

On the other hand, in Cuyín Manzano, 87 species were recorded, of which 47 were native and 40 exotic. Of the total number of species, 63 (72%) were cited for medicinal use and 24 (28%) were mentioned for edible purposes [20].

## Discussion

In the present study we have found that traditional ecological knowledge related to horticultural practice and wild plant gathering is still transmitted over generations in rural populations of North-western Patagonia.

In the case of the Pilcaniyeu community, the transmission of traditional knowledge related to horticultural practices begins in the early stages of childhood, when accompanying their parents to cultivate the land. In this family tradition, women seem to play a predominant role. This knowledge is mostly transmitted vertically through family dissemination. In the Cuyin Manzano community, wild plant gathering is transmitted by family members during childhood as well. Vertical transmission is the main mode of acquiring this traditional knowledge. Mothers are the first mentioned as principal transmitters, this fundamental role of women has also been found in other studies [26-28]. For example, it has been observed that wild fruit gathering is an activity mainly conducted by women, who transmit not only their traditional knowledge but also certain values of respect and connection with nature, generation after generation [29]. Similarly, women are generally the ones in charge of home-gardens, as well as of the transmission of traditional knowledge related to horticultural practices (e.g. following moon cycles, elaboration of natural herbicides, collection of seeds) [3,30,31]. Moreover, other studies emphasize female participation in projects destined to sustainable management practices in forests, due to their thorough knowledge of the area [32].

In Pilcaniyeu, people recognized and valued their personal experience related to horticultural practices. However, horizontal transmission is relevant at present, i.e. horticultural learning continues during adulthood. At this life time, which has significant extra-familiar contributions, locals interchange knowledge and practices, probably relearning and changing their previously acquired information. This knowledge may or may not agree with what they learnt as a family tradition. Horizontal mode of transmission related to home-garden management was also observed in other communities [31]. Moreover, extension agents have introduced new practices and technology through a "one-toward-many" mode of transmission. According to Boesch & Tomasello [6] cultural innovations may be rapidly incorporated by a community when these are introduced by an influential group. This could be the case of the Pilcaniyeu community where extension agents might have initiated cultural changes on horticultural practices. The use of greenhouses was first introduced as a new technology by extension agents, and afterwards it spread among locals through horizontal transmission. This could be an example of multiple sources of information and knowledge which coexist within a group, suggesting diverse ways of unfolding social learning processes [33].

The fact that horizontal transmission was not conspicuously detected in Cuyin Manzano could be related to methodological biases associated with self-report means of inquiry. When interviews rely on self-reports, it is possible that informants tend to overemphasize the role of parents in acquiring ethnobotanical knowledge [7]. During childhood, this knowledge is learned through vertical transmission although, later in life, locals often acquire extra-familiar information without being conscious of this process. This might have also occurred in the Pilcaniyeu community in spite of the overt influence received from outsiders. Therefore, horizontal inputs might have been underestimated in the collected data, an issue that should be kept into consideration. As an example, they minimized the importance of external agents in the interviews, although most of families have incorporated greenhouses provided by them.

The incorporation of new practices and technology, which could lead to innovative processes, could also imply the loss of traditional ecological knowledge. At the same time, it could allow for the integration of new and ancestral practices, generating hybrid knowledge [34]. This process might indicate a dynamic response to their changing living conditions, suggesting local capacity for building social-ecological resilience [10]. Resilience implies the capacity for learning and adapting [8]. This process might be occurring in Pilcaniyeu, given that 68.6% of the families incorporated greenhouse, thus integrating their ancestral knowledge with new technologies and knowledge. Furthermore, the cultivation of exotic species and the replacement of native ones, which started with the Spanish Conquest [12], has become a long standing practice that still occurs nowadays. This process, which began in the Mapuche ancestral habitat, the forest, appears to have been enhanced in this new arid context, with the incorporation, for example, of greenhouses. This might indicate further adaptive possibilities.

Moreover, in Cuyin Manzano resilience could also be observed in relation to wild exotic species gathering. Though wild exotic plants were observed many years ago in this region [24], the relatively high proportion of non native species use could also be indicating a longstanding process of change [16]. Both local practices, i.e. wild plant gathering and horticulture, are undergoing several transformations, showing the influences of environmental changes and cultural transmission.

Both wild plant gathering and horticultural practices imply personal connections with nature that might provide opportunities for learning, encouraging the development of attitudes which are associated with the protection of the environment [36]. At present, there are fewer opportunities, especially for children, to spend time with and learn from parents, grandparents and others who are knowledgeable about conservation practices and beliefs [10], in addition to the introduction of schooling into family life [3]. Local knowledge is remembered through social memory, which describes how an individual thought or observation can become part of the collective knowledge of a group, so as to allow communal understanding of environmental change and the transmission of experience [11].

## Conclusion

In order to preserve traditional ecological knowledge, it would be convenient to generate connectedness within networks and groups [33,37] so as to encourage vertical and horizontal transmission of traditional practices among locals. Therefore, it could be useful to bring back these results to the rural people and to promote community participation activities tending to share traditional knowledge such as workshops, field trips and seed interchange [31]. This could enable greater integration between local and scientific knowledge, allowing for cultural and biological diversity. For this purpose, it would also be necessary to create new mechanisms of communication between locals and western Institutions tending to facilitate better comprehension and self respect [33]. In this way, investigations such as the present study could help by forming a bridge between local dwellers and extension agents who want to promote rural development. We consider that being receptive to local people's traditional ecological knowledge is important in order to generate more efficient aid to rural communities, as well as fostering western culture.

## Competing interests

The authors declare that they have no competing interests.

## Authors' contributions

CE carried out the field work and acquisition of data. AL and ML made substancial contributions to analysis and interpretation of data. They also have been involved in drafting the manuscript, revising it critically and giving final approval of the version to be published. All authors read and approved the final manuscript.

## Supplementary Material

Additional file 1 Plant species collected in home-gardens, greenhouses and gardens utilized by Pilcaniyeu people. Click here for file
