# Supplementary material for: Cultural Transmission of Traditional Knowledge in two populations of North-western Patagonia
Source: J Ethnobiol Ethnomed. 2008 Dec 15;4:25. doi: 10.1186/1746-4269-4-25 (PMC2614966; doi:10.1186/1746-4269-4-25)
Supplement: Additional file 1 — Plant species collected in home-gardens, greenhouses and gardens utilized by Pilcaniyeu people. [file 1746-4269-4-25-S1.doc]

# Additional files

## Additional file 1 -Plant species collected in home-gradens, greenhouses and gardens utilized by Pilcaniyeu people.

Botanic Family

| Common name | *Scientific name* | Origin |
| --- | --- | --- |

# Amaryllidaceae

| Narcizo | *Narcissus* sp. | Exotic |
| --- | --- | --- |
| Anacardiaceae Molle | *Schinus o´donellii* Barkley | Native |
| Apiaceae Apio | *Apium graveolens* L. | Exotic |
| Cilantro | *Coriandrum sativum* L. | Exotic |
| Hinojo | *Foeniculum vulgare* Mill. | Exotic |
| Perejil | *Petroselinum crispum* (Mill.) Nym. | Exotic |
| Perejil crespo | *Petroselinum crispum* var. *crispum* (Mill.) Airy-Shaw | Exotic |
| Zanahoria | *Daucus carota* var. *sativa* L. | Exotic |
| Araliaceae |  |  |
| Hiedra | *Hedera helix* L. | Exotic |
| **Araucariaceae** |  |  |
| Araucaria | *Araucaria araucana* (Mol.) K. Koch | Native |
| Asteraceae Achicoria | *Taraxacum officinale* Web. | Exotic |
| Ajenjo | *Artemisia absinthium* L. | Exotic |
| Caléndula | *Calendula officinalis* L. | Exotic |
| Carqueja | *Baccharis sagittalis* (Less.) DC. | Native |
| Chilca | *Baccharis salicifolia* (Ruiz & Pav.) Pers. | Native |
| Coqueta | *Bellis perennis* L. | Exotic |
| Éter | *Artemisia abrotanum* L. | Exotic |
| Girasol enano | *Helianthus tuberosus* L. | Exotic |
| Lechuga | *Lactuca sativa* L. | Exotic |
| Manzanilla | *Chrysanthemum parthenium* ( L). Bernhardi | Exotic |
| Margarita | *Chrysanthemum sp.* | Exotic |
| Margarita | *Matricaria inodora* L. | Exotic |
| Milenrama | *Achillea millefolium* L. | Exotic |
| Palma | *Tanacetum vulgare* L. | Exotic |
| Yerba de San Juan  menta extranjera o turca | *Tanacetum balsamita* L. | Exotic |
| Brassicaceae Alelí | *Cheirantus cheiri* L. | Exotic |
| Matuerzo | *Coronopus didymus* (L.) Smith | Exotic |
| Nabiza | *Brasicca napus* var. *arvensis* f. *annus* (Schubl. et Mart.) Thell | Exotic |
| Nabo | *Brassica rapa* L. | Exotic |
| Rabanito | *Raphanus sativus* L. | Exotic |
| Repollo | *Brassica oleraceae* var. *capitata* L. | Exotic |
| Repollo corazón de buey | *Brassica oleraceae* L. | Exotic |
| Buddlejaceae Pañil | *Buddleja araucana* Phil. | Native |
| Caprifoliaceae Sauco | *Sambucus nigra* L. | Exotic |
| Caryophyllaceae Clavel | *Dianthus caryophyllus* L. | Exotic |
| Celastraceae Maitén | *Maytenus boaria* Molina | Native |
| Chenopodiaceae Acelga | *Beta vulgaris* var. *cicla* L. | Exotic |
| Espinaca | *Spinacia oleracea* L. | Exotic |
| Paico | *Chenopodium ambrosioides* L. | Native |
| Remolacha | *Beta vulgaris* var. *rapacea* L. | Exotic |
| Crassulaceae Rochela | (?) | Exotic |
| Cucurbitaceae Melón | *Cucumis melo* L. | Exotic |
| Pepino | *Cucumis sativus* L. | Exotic |
| Zapallito | *Cucurbita maxima* var. *zapallito* (Carr.) Millán | Exotic |
| Zapallito zuchini | *Cucurbita pepo* convar. *giromontiina* Duch | Exotic |
| Zapallo | *Cucurbita pepo* L. | Exotic |
| **Elaeagnaceae**  Olivillo | *Elaeagnus angustifolia* L. | Exotic |
| Enoteraceae Clarquea | *Clarkia elegans* Dougl. | Exotic |
| Godesia | *Godetia* sp. | Exotic |
| Fabaceae Alfalfa | *Medicago sativa* L. | Exotic |
| Arveja | *Pisum sativum* L. | Exotic |
| Chocho | *Lupinus arboreus* Sims. | Exotic |
| Clarín | *Lathyrus latifolius* L. | Exotic |
| Haba | *Vicia faba* L. | Exotic |
| Monte guanaco | *Anarthrophyllum rigidum* (Gilies ex Hook. & Arn.) Hieron. | Native |
| Paramela | *Adesmia boronioides* Hook.f. | Native |
| Poroto | *Phaseolus* sp. | Exotic |
| Retama | *Cytisus scoparius* L. (Link.) | Exotic |
| Vicia | *Vicia* sp. | Exotic |
| **Geraniaceae** |  |  |
| Malvón | *Pelargonium* sp. | Exotic |
| Lamiaceae Albahaca | *Ocimum basilicum* L. | Exotic |
| Lavanda | *Lavandula* sp. | Exotic |
| Malva rubia | *Marrubium vulgare* L. | Exotic |
| Menta blanca | *Mentha rotundifolia* (L.) Huds. | Exotic |
| Menta negra | *Mentha spicata* L. | Exotic |
| Orégano | *Origanum vulgare* L. | Exotic |
| Chascudo | *Origanum* (?) | Exotic |
| Poleo | *Mentha pulegium* L. | Exotic |
| Romero | *Rosmarinus officinalis* L. | Exotic |
| Salvia | *Salvia officinalis* L. | Exotic |
| Té del gato | *Nepeta cataria* L. | Exotic |
| Tomillo | *Thymus vulgaris* L. | Exotic |
| Toronjil | *Melisa officinalis* L. | Exotic |
| Lauraceae Laurel | *Laurus nobilis* L. | Exotic |
| Liliaceae Ajo | *Allium sativum* L. | Exotic |
| Ajo puerro | *Allium porrum* L. | Exotic |
| Cebolla | *Allium cepa* L. | Exotic |
| Cebolla de verdeo | *Allium fistulosum* L. | Exotic |
| Cebollita | *Allium* sp. | Exotic |
| Chalota | *Allium schoenoprasum* L. | Exotic |
| Tulipán | *Tulipa* sp. | Exotic |
| Linaceae Lino | *Linum usitatissimum* L. | Exotic |
| Malvaceae Malva | *Malva sylvestris* L. | Exotic |
| Malvón | *Althaea officinalis* L. | Exotic |
| Oleaceae Lila | *Syringa vulgaris* L. | Exotic |
| Papaveraceae Amapola | *Papaver rhoeas* L. | Exotic |
| Celedonia | *Chelidonium majus* L. | Exotic |
| Copa de oro | *Eschscholtzia californica* Cham. | Exotic |
| Pinaceae Pino | *Pinus* sp. | Exotic |
| Plantaginaceae Llantén | *Plantago lanceolata* L. | Exotic |
| Poaceae Maíz | *Zea mays* L. | Exotic |
| Poa | *Poa* sp | ? |
| Polygonaceae Ruibarbo | *Rheum rhabarbarum L.* | Exotic |
| Ranunculaceae Espuela de caballero o |  |  |
| Pajarito | *Consolida ajacis (*L.) Schur | Exotic |
| Rosaceae Cerezo | *Prunus avium* L. | Exotic |
| Ciruelo | *Prunus domestica* L. | Exotic |
| Corona de novia | *Spiraea lanceolata* Poir. | Exotic |
| Duraznero | *Prunus persica* L. Batsch | Exotic |
| Frambuesa | *Rubus idaeus* L. | Exotic |
| Frutilla | *Fragaria vesca* L. | Exotic |
| Guindo | *Prunus cerasus* L. | Exotic |
| Manzano | *Malus domestica* Borkh | Exotic |
| Membrillo | *Cydonia oblonga Mill.* | Exotic |
| Níspero | *Mespilus germanica* L. | Exotic |
| Rosa | *Rosa* sp. | Exotic |
| Rutaceae Naranjo | *Citrus sinensis* L. | Exotic |
| Ruda | *Ruta graveolens* L. | Exotic |
| Salicaceae Álamo | *Populus nigra* L. | Exotic |
| Álamo plateado | *Populus alba* L. | Exotic |
| Sauce | *Salix* sp. | Exotic |
| Saxifragaceae Casis | *Ribes* sp. | Exotic |
| Corinto | *Ribes aureum* Pursh. | Exotic |
| Grosella | *Ribes grossularia* L. | Exotic |
| Zarzaparrilla | *Ribes magellanicum* Poir. | Native |
| Scrophulariaceae Conejito | *Antirrhinum majus* L. | Exotic |
| Solanaceae Morrón | *Capsicum annuum* L. | Exotic |
| Papa | *Solanum tuberosum* L. | Exotic |
| Tomate | *Lycopersicum esculentum* var. *esculentum* Mill. | Exotic |
| Ulmaceae Olmo | *Ulmus* sp. | Exotic |
| Urticaceae Ortiga | *Urtica dioica* L. | Exotic |
| Verbenaceae Retamo | *Diostea juncea* (Gillies & Hook) Miers. | Native |
| Violaceae Pensamiento | *Viola tricolor* L. | Exotic |
| Vitaceae Uva | *Vitis vinifera* L. | Exotic |
|  |  |  |
|  |  |  |
